# Combination of supported bimetallic rhodium–molybdenum catalyst and cerium oxide for hydrogenation of amide

**DOI:** 10.1088/1468-6996/16/1/014901

**Published:** 2015-01-13

**Authors:** Yoshinao Nakagawa, Riku Tamura, Masazumi Tamura, Keiichi Tomishige

**Affiliations:** Department of Applied Chemistry, School of Engineering, Tohoku University, 6-6-07, Aoba, Aramaki, Aoba-ku, Sendai 980-8579, Japan

**Keywords:** heterogeneous catalysis, rhodium, ceria, amide, amine

## Abstract

Hydrogenation of cyclohexanecarboxamide to aminomethylcyclohexane was conducted with silica-supported bimetallic catalysts composed of noble metal and group 6–7 elements. The combination of rhodium and molybdenum with molar ratio of 1:1 showed the highest activity. The effect of addition of various metal oxides was investigated on the catalysis of Rh–MoO_*x*_/SiO_2_, and the addition of CeO_2_ much increased the activity and selectivity. Higher hydrogen pressure and higher reaction temperature in the tested range of 2–8 MPa and 393–433 K, respectively, were favorable in view of both activity and selectivity. The highest yield of aminomethylcyclohexane obtained over Rh–MoO_*x*_/SiO_2_ + CeO_2_ was 63%. The effect of CeO_2_ addition was highest when CeO_2_ was not calcined, and CeO_2_ calcined at >773 K showed a smaller effect. The use of CeO_2_ as a support rather decreased the activity in comparison with Rh–MoO_*x*_/SiO_2_. The weakly-basic nature of CeO_2_ additive can affect the surface structure of Rh–MoO_*x*_/SiO_2_, i.e. reducing the ratio of Mo–OH/Mo–O^−^ sites.

## 1. Introduction

Heterogeneous catalysis is one of the important applications of inorganic materials [1, 2]. Reduction reactions are one class of target reactions for heterogeneous catalysis and are widely used in both laboratory-scale organic synthesis and industrial processes [3–5]. Hydrogenation of amides produces amines that have been used in various fields such as pharmaceutical industries (equation (1)) [6, 7]. However, this reaction is rather difficult than other reduction reactions of carbonyl compounds, because the *π*-electrons in the carbonyl groups of amides are stabilized by the conjugation with nitrogen (equation (2)). Possible formation of various by-products such as alcohols, ammonia and secondary amines is another difficulty.
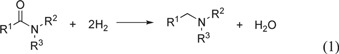


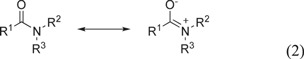



Conventionally, reduction of amides has been conducted non-catalytically with strongly reductive reagents such as LiAlH_4_. Problems with this conventional method include the difficult handling and cost of the reactive reagents and complex workup. Therefore, development of catalytic systems for hydrogenation of amides with molecular hydrogen has been intensively carried out. Homogeneous systems using Ru complex catalysts have been reported to be effective [8–12]; however, homogeneous systems have difficulty in removal of catalyst from the reaction mixture after use. Heterogeneous catalysts are favorable in view of workup. Several research groups have reported that unsupported bimetallic catalysts which consist of noble metal and group 6 or 7 elements are effective such as Rh–Mo, Rh–Re, Ru–Re and Pt–Re [13–16]. However, reports of effective supported catalysts are limited [13, 17–19], while for other reduction reactions supported catalysts are more common than unsupported ones. Recently, we have showed that silica- or carbon-supported bimetallic catalysts composed of noble metal and group 6–7 elements are very effective for various reduction reactions such as hydrogenation of unsaturated aldehydes [20–22], hydrogenolysis of poly-alcohols [23–27] and hydrogenation of carboxylic acids [28–30]. We have also showed that the performance of some catalysts in this category is affected by addition of solid metal oxide in the reaction media [31–33]: for example, hydrogenolysis activity of Ir–ReO_*x*_/SiO_2_ catalyst is promoted by addition of solid acid such as H-ZSM-5 zeolite or silica-alumina and decreased by addition of basic oxides such as CeO_2_ [31]. In this study, we applied various silica-supported bimetallic catalysts combined with metal oxides to hydrogenation of amide. We found that the addition of CeO_2_ much increases the activity of Rh–MoO_*x*_/SiO_2_ catalyst.

## 2. Experimental

*M*^1^–*M*^2^O_*x*_/SiO_2_ catalysts (*M*^1^ = noble metal; *M*^2^ = Mo, W and Re) were prepared by sequential impregnation method as reported previously [23–26]. First, *M*^1^/SiO_2_ catalysts were prepared by impregnating SiO_2_ (Fuji Silysia G-6; BET surface area 535 m^2^ g^−1^) with an aqueous solution of noble metal precursor (RhCl_3_ · 3H_2_O, H_2_PtCl_6_ · 6H_2_O, RuCl_3_ · *n*H_2_O, PdCl_2_ and H_2_IrCl_6_). The loading amount of *M*^1^ was 4 wt%. After impregnation, they were dried at 383 K overnight. And then the second impregnation was conducted with an aqueous solution of *M*^2^ precursor ((NH_4_)_6_Mo_7_O_24_ · 4H_2_O, (NH_4_)_10_W_12_O_41_ · 5H_2_O and NH_4_ReO_4_) to prepare *M*^1^–*M*^2^O_*x*_/SiO_2_. The loading amount of *M*^2^ was set to *M*^2^/*M*^1^ = 1 in molar basis unless noted. After impregnation, the bimetallic catalysts were dried at 383 K overnight and calcined at 773 K for 3 h. Monometallic catalysts were also calcined at 773 K for 3 h when used for catalytic reaction.

Activity tests were performed in a 190 mL stainless steel autoclave with an inserted glass vessel. Typically, catalyst (100 mg), cyclohexanecarboxamide (0.25 g; 2 mmol), 1,2-dimethoxyethane (solvent, 20 g) and CeO_2_ (Daiichi Kigenso HS, 120 m^2^ g^−1^; 100 mg) were put into an autoclave together with a spinner. After sealing the reactor, the air content was quickly purged by flushing three times with 1 MPa hydrogen. The autoclave was then heated to reaction temperature (typically 413 K), and the temperature was monitored using a thermocouple inserted in the autoclave. After the temperature reached the desired one, the H_2_ pressure was increased to set value (typically 8 MPa). During the experiment, the stirring rate was fixed at 500 rpm (magnetic stirring). After appropriate reaction time (typically 4 or 24 h), the autoclave was quickly cooled to room temperature, and the gases were collected in a gas bag. *n*-dodecane (0.1 mL) was added to the liquid content as an internal standard material, and the catalyst was separated by filtration. The products in gas and liquid phases were analyzed with GC and GC-MS. A CP-Sil-5 capillary column was used for separation. The formation of gaseous products was always negligible. Selectivities were calculated based on the number of carbon atoms. The reproducibility of carbon balance in different runs with the same conditions was ±3%. The loss of carbon balance was included to ‘others’. Other metal oxides used instead of CeO_2_ were ZrO_2_ (Daiichi Kigenso; 88 m^2^ g^−1^), TiO_2_ (AEROXIDE P25; 50 m^2^ g^−1^), *γ*-Al_2_O_3_ (Sumitomo KHO-24; 140 m^2^ g^−1^), MgO (Ube 500 A; 33 m^2^ g^−1^), SiO_2_–Al_2_O_3_ (JGC C&C and Catalysis Society of Japan, JRC-SAL-3; 504 m^2^ g^−1^), and H-ZSM-5 (Süd Chemie and Catalysis Society of Japan, JRC-25–90 H; 325 m^2^ g^−1^).

## 3. Results and discussion

### 3.1. Hydrogenation of cyclohexanecarboxamide over various catalysts

First, we applied various silica-supported bimetallic catalysts to hydrogenation of cyclohexanecarboxamide (CyCONH_2_) (table 1). We chose cyclohexanecarboxamide as a representative substrate of primary amide [14, 18], and the target product of this reaction is aminomethylcyclohexane (CyCH_2_NH_2_). By-products include cyclohexanemethanol (CyCH_2_OH) which can be formed by C–N dissociation of amide, cyclohexanecarboxylic acid (CyCOOH) which is produced by hydrolysis of cyclohexanecarboxamide, and bis(cyclohexylmethyl)amine ((CyCH_2_)_2_NH; secondary amine). The formation mechanism of bis(cyclohexylmethyl)amine is discussed in section 3.5. Significant loss of carbon balance was observed in many cases. We included the loss to the selectivity to ‘others’ because TG analysis confirmed the deposition of organic material on the catalyst. Rh–MoO_*x*_/SiO_2_ showed the highest activity and selectivity to aminomethylcyclohexane in *M*^1^–MoO_*x*_/SiO_2_ catalysts (*M*^1^ = noble metal) and Rh–*M*^2^O_*x*_/SiO_2_ catalysts (*M*^2^ = Mo, W and Re). Monometallic Rh/SiO_2_ and MoO_*x*_/SiO_2_ catalysts showed almost no activity in amine formation. The effect of Mo addition to Rh/SiO_2_ catalyst is more evident than in the reported case of unsupported Rh–Mo catalysts where monometallic Rh catalyst shows some activity [13]. Among Rh–MoO_*x*_/SiO_2_ catalysts with different Mo/Rh ratios, the catalyst with Mo/Rh = 1 showed the highest activity. The catalysts with lower Mo amount showed higher selectivity to secondary amine in addition to lower activity. This activity trend is different from that of the same catalysts in C–O hydrogenolysis [24, 25, 34] and amino acid hydrogenation [29]. The catalyst with Mo/Rh = 1/8 shows the highest activity in C–O hydrogenolysis and amino acid hydrogenation. There may be difference in the active sites between amide hydrogenation and these reactions. We selected Rh–MoO_*x*_/SiO_2_ (Mo/Rh = 1) in the following studies.

**Table 1.** Hydrogenation of cyclohexanecarboxamide over various catalysts^a^.

Entry	Catalyst	Molar ratio of *M*^2^/noble metal	Conv. (%)	Selectivity (%)				
				CyCH_2_NH_2_	CyCH_2_OH	(CyCH_2_)_2_NH	CyCOOH	Others
1	Rh–MoO_*x*_/SiO_2_	1	74	43	6	30	<1	21^b^
2	Pt–MoO_*x*_/SiO_2_	1	<1	—	—	—	—	—
3	Ru–MoO_*x*_/SiO_2_	1	1^c^	∼15	∼30	∼55	<5	<5
4	Pd–MoO_*x*_/SiO_2_	1	1^c^	∼10	∼30	∼60	<5	<5
5	Ir–MoO_*x*_/SiO_2_	1	12	<1	<1	<1	<1	>99^b^
6	Rh–WO_*x*_/SiO_2_	1	3^c^	∼20	∼5	∼70	<5	<5
7	Rh–ReO_*x*_/SiO_2_	1	20	47	4	39	<1	10^b^
8	Rh–MoO_*x*_/SiO_2_	0.25	29	23	5	50	<1	21^b^
9	Rh–MoO_*x*_/SiO_2_	0.5	58	41	5	36	<1	19^b^
10	Rh–MoO_*x*_/SiO_2_	2	67	44	8	28	<1	21^b^
11	Rh/SiO_2_	0	2^c^	∼15	∼30	∼55	<5	<5
12	MoO_*x*_/SiO_2_^d^	—	24	<1	<1	<1	<1	>99^b^

a Reaction conditions: catalyst 100 mg (noble metal 4 wt%), CyCONH_2_ 0.25 g (2 mmol), 1,2-dimethoxyethane 20 g, H_2_ 8 MPa, 413 K, 24 h. Cy = cyclohexyl.

b Representing loss of carbon balance predominantly via formation of solid products on the catalyst surface.

c Selectivities in these entries are nominal ones as the low conversions preclude obtained data from being comparable with other entries.

d Mo 3.7 wt%.

### 3.2. Addition of metal oxides to the catalytic system of Rh–MoO_*x*_/SiO_2_

We investigated the effects of addition of metal oxides on the catalysis of Rh–MoO_*x*_/SiO_2_ (Mo/Rh = 1). The results are shown in table 2. The reaction time was set to be shorter than table 1 to compare the activities. The selectivities of Rh–MoO_*x*_/SiO_2_ were almost the same at different reaction times (table 1, entry 1; table 2, entry 8). Addition of weakly basic CeO_2_ and *γ*-Al_2_O_3_ increased the activity (conversion of substrate) and selectivity to CyCH_2_NH_2_. The formation of secondary amine ((CyCH_2_)_2_NH) was significantly suppressed by the addition. The addition of CeO_2_ showed the best effect. On the other hand, acidic additives such as H-ZSM-5, silica-alumina, ZrO_2_ and TiO_2_ showed little effect on the conversion and much decreased the selectivity to CyCH_2_NH_2_. Strongly basic MgO also decreased the selectivity to CyCH_2_NH_2_ and had little effect on the conversion. The selectivity to ‘others’, which comprised solid polymerized products deposited on the catalyst, was increased by addition of acidic or strongly basic oxides.

**Table 2.** Hydrogenation of cyclohexanecarboxamide over Rh–MoO_*x*_/SiO_2_ catalyst + various metal oxides^a^.

Entry	Metal oxide	Conv. (%)	Selectivity (%)				
			CyCH_2_NH_2_	CyCH_2_OH	(CyCH_2_)_2_NH	CyCOOH	Others^b^
1	CeO_2_	89	62	6	5	<1	27
2	ZrO_2_	26	21	5	11	26	37
3	TiO_2_	24	21	5	15	20	40
4	*γ*-Al_2_O_3_	61	53	4	8	<1	35
5	MgO	20	39	4	15	<1	42
6	SiO_2_–Al_2_O_3_	27	28	5	14	<1	53
7	H-ZSM-5	34	17	3	11	<1	69
8	None	18	49	7	26	<1	19

a Reaction conditions: Rh–MoO_*x*_/SiO_2_ 100 mg (Rh 4 wt%, Mo/Rh = 1), CyCONH_2_ 0.25 g (2 mmol), metal oxide 100 mg, 1,2-dimethoxyethane 20 g, H_2_ 8 MPa, 413 K, 4 h. Cy = cyclohexyl.

b Representing loss of carbon balance predominantly via formation of solid products on the catalyst surface.

**Figure 1.**
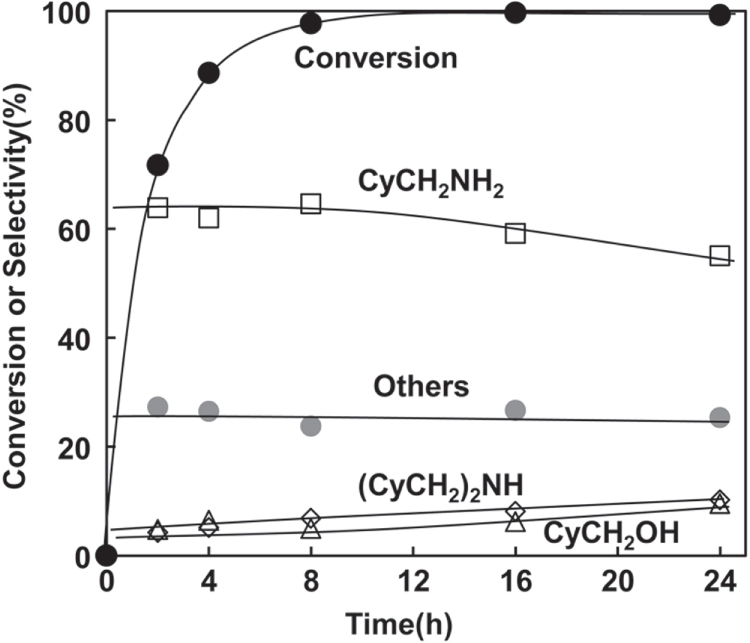
Time course of hydrogenation of cyclohexanecarboxamide (CyCONH_2_) over Rh–MoO_*x*_/SiO_2_ + CeO_2_. Reaction conditions: Rh–MoO_*x*_/SiO_2_ (Rh 4 wt%, Mo/Rh = 1) 100 mg, CeO_2_ (uncalcined) 100 mg, 1,2-dimethoxyethane 20 g, H_2_ 8 MPa, 413 K. Cy = cyclohexyl. ‘Others’ comprise unknown solid products leading to loss of carbon balance during catalysis.

Figure 1 shows the time course of hydrogenation of CyCONH_2_ over Rh–MoO_*x*_/SiO_2_ in combination with CeO_2_. The selectivities were hardly changed until the total conversion of CyCONH_2_, and then the selectivity to CyCH_2_NH_2_ was gradually decreased and that to (CyCH_2_)_2_NH was gradually increased. The highest yield of CyCH_2_NH_2_ was 63% obtained at 8 h (equation (3)). Although the yield value was lower than that over unsupported Rh/Mo catalyst in the literature (87%), the activity was significantly higher (CyCONH_2_/Rh_total_ = 50 at 413 K, 8 h in this study; CyCONH_2_/Rh_total_ = 20 at 433 K, 16 h in the literature [13]).
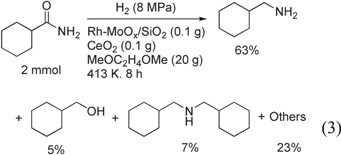



The life of the catalyst is also an issue. The reusability of Rh–Mo catalysts has been reported for reduction reactions [13, 24, 30], and good stability in the structure has been observed by XRD and EXAFS analyses [24, 30]. However, the deposition of organic material on the catalyst in this system clearly limits the long-term use. The development of effective regeneration method without aggregation of active metal particles is a target of further study.

### 3.3. Effect of amount and type of CeO_2_ additive

**Figure 2.**
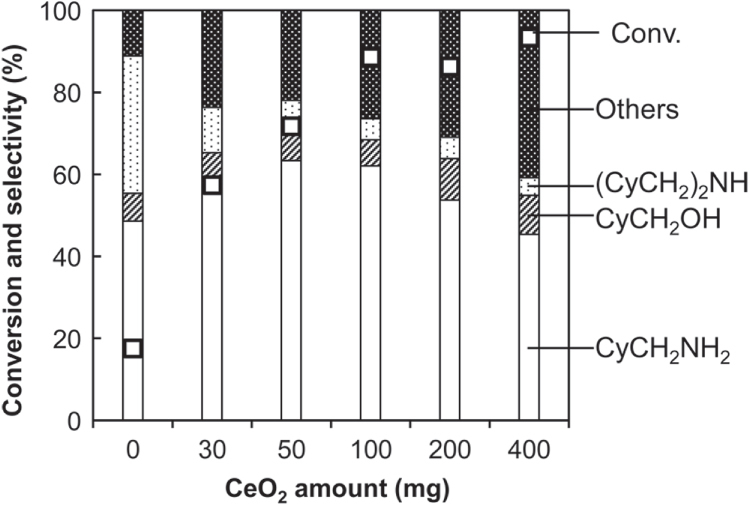
Hydrogenation of cyclohexanecarboxamide (CyCONH_2_) over Rh–MoO_*x*_/SiO_2_ + various amounts of CeO_2_. Reaction conditions: Rh–MoO_*x*_/SiO_2_ (Rh 4 wt%, Mo/Rh = 1) 100 mg, CeO_2_ (uncalcined) 0–400 mg, 1,2-dimethoxyethane 20 g, H_2_ 8 MPa, 413 K, 4 h. Cy = cyclohexyl. ‘Others’ comprise unknown solid products leading to loss of carbon balance during catalysis.

The effect of amount of CeO_2_ additive on the catalysis is shown in figure 2. The activity was increased with increasing CeO_2_ amount; however, too much amount of CeO_2_ decreased the selectivity to CyCH_2_NH_2_ and increased the selectivity to unknown by-products. 50–100 mg of CeO_2_ was the best amount as additive to Rh–MoO_*x*_/SiO_2_, and we used 100 mg of CeO_2_ in the other parts of this study.

**Figure 3.**
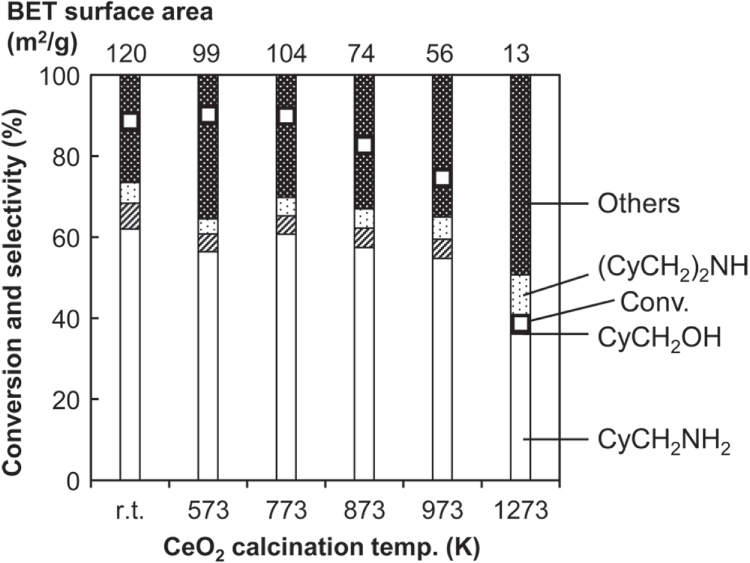
Hydrogenation of cyclohexanecarboxamide (CyCONH_2_) over Rh–MoO_*x*_/SiO_2_ + calcined CeO_2_. Reaction conditions: Rh–MoO_*x*_/SiO_2_ (Rh 4 wt%, Mo/Rh = 1) 100 mg, CeO_2_ 100 mg, 1,2-dimethoxyethane 20 g, H_2_ 8 MPa, 413 K, 4 h. Cy = cyclohexyl. ‘Others’ comprise unknown solid products leading to loss of carbon balance during catalysis. ‘r.t.’ stands for room temperature.

While we used commercial CeO_2_ without calcination pretreatment, it is well known that the crystallinity and the surface area of CeO_2_ can be changed by calcination pretreatment [35, 36]. The surface area is reduced by calcination at higher temperature, and the surface of CeO_2_ samples without calcination or calcined at lower temperature (<873 K) is partly amorphous [37]. Indeed, we have used CeO_2_ catalysts after calcination at different temperatures for various CO_2_ utilization reactions such as carbonate synthesis, and we have found that CeO_2_ after 873 K calcination shows the highest activity probably because crystalline CeO_2_ surface is the active site [37–40]. Figure 3 shows the results of hydrogenation of CyCONH_2_ over Rh–MoO_*x*_/SiO_2_ and CeO_2_ calcined at various temperatures. The addition effect of CeO_2_ was highest when CeO_2_ was not calcined or calcined at <773 K, and the effect became smaller when CeO_2_ was calcined at higher temperature. This behavior shows that the addition effect was mostly determined by the surface area.

**Figure 4.**
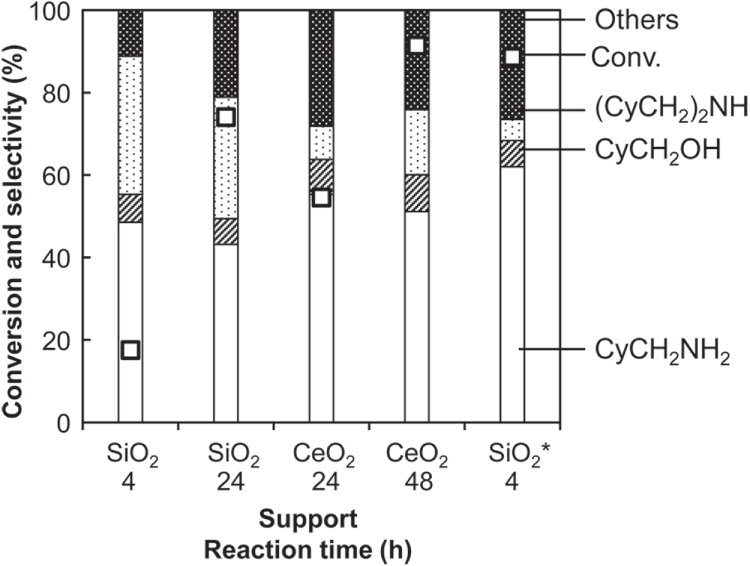
Hydrogenation of cyclohexanecarboxamide (CyCONH_2_) over Rh–MoO_*x*_ catalyst with different supports. Reaction conditions: Rh–MoO_*x*_/support (Rh 4 wt%, Mo/Rh = 1) 100 mg, 1,2-dimethoxyethane 20 g, H_2_ 8 MPa, 413 K, 4–48 h. Cy = cyclohexyl. ‘Others’ comprise unknown solid products leading to loss of carbon balance during catalysis. ∗: CeO_2_ (100mg).

We also prepared CeO_2_-supported Rh–MoO_*x*_ catalyst. However, the catalytic activity was even lower than Rh–MoO_*x*_/SiO_2_ without CeO_2_ addition, although the dimerization side-reaction was surely suppressed similarly to external addition of CeO_2_ (figure 4). These data suggest that the direct interaction between CeO_2_ and Rh (or Mo) is not important in the catalysis.

### 3.4. Effect of reaction conditions

**Figure 5.**
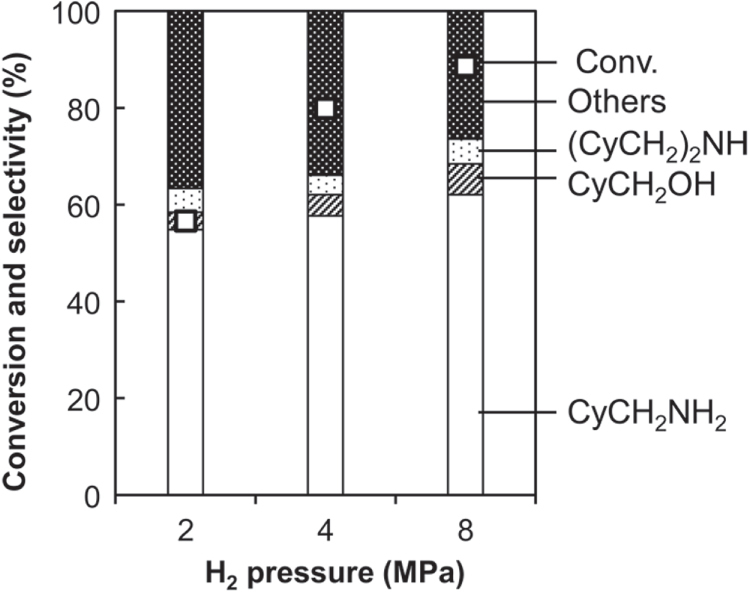
Effect of H_2_ pressure on hydrogenation of cyclohexanecarboxamide (CyCONH_2_) over Rh–MoO_*x*_ catalyst + CeO_2_. Reaction conditions: Rh–MoO_*x*_/SiO_2_ (Rh 4 wt%, Mo/Rh = 1) 100 mg, CeO_2_ (uncalcined) 100 mg, 1,2-dimethoxyethane 20 g, H_2_ 2–8 MPa, 413 K, 4 h. Cy = cyclohexyl. ‘Others’ comprise unknown solid products leading to loss of carbon balance during catalysis.

The effect of hydrogen pressure on the catalysis of Rh–MoO_*x*_/SiO_2_ + CeO_2_ was examined (figure 5). It should be noted that comparison of selectivities at different conversion level is possible because selectivities are hardly changed on reaction time until complete conversion (figure 1). Higher activity was observed under higher hydrogen pressure. The selectivity to CyCH_2_NH_2_ was also slightly increased with increasing hydrogen pressure, and instead the formation of unknown by-products was suppressed.

**Figure 6.**
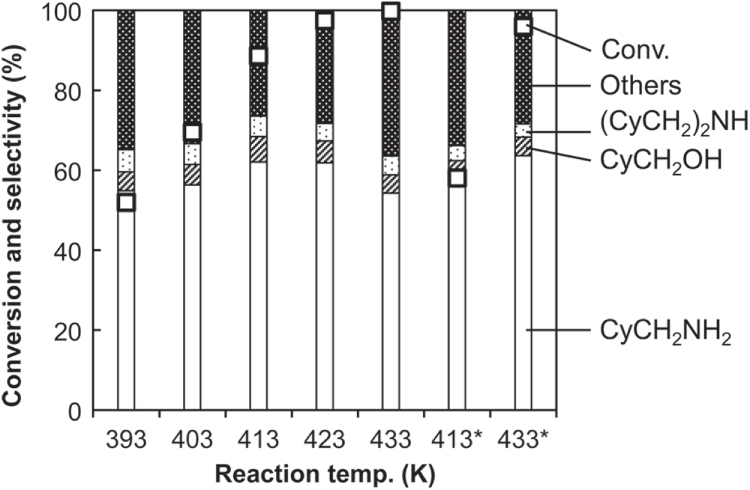
Effect of reaction temperature on hydrogenation of cyclohexanecarboxamide (CyCONH_2_) over Rh–MoO_*x*_ catalyst + CeO_2_. Reaction conditions: Rh–MoO_*x*_/SiO_2_ (Rh 4 wt%, Mo/Rh = 1) 100 mg, CeO_2_ (uncalcined) 100 mg, 1,2-dimethoxyethane 20 g, H_2_ 8 MPa, 393–433 K, 4 h. Cy = cyclohexyl. ‘Others’ comprise unknown solid products leading to loss of carbon balance during catalysis. ∗: half amount of catalyst and CeO_2_ was used (50 mg each).

Figure 6 shows the effect of reaction temperature. Higher temperature increased the activity. The selectivity to CyCH_2_NH_2_ became slightly higher with increasing the temperature, as clearly seen up to 423 K. At 433 K, the conversion level was too high to compare selectivity in the standard reaction conditions. Therefore we further conducted reaction tests at 433 K (and 413 K for comparison) with smaller amount of Rh–MoO_*x*_/SiO_2_ catalyst and CeO_2_. The selectivity to aminomethylcyclohexane was higher at 433 K than 413 K. After all, higher hydrogen pressure and higher reaction temperature are favorable in this reaction.

### 3.5. Reaction mechanism

We have characterized Rh–MoO_*x*_/SiO_2_ catalysts with various Mo/Rh ratios (0.13−0.5) in the previous papers [24, 34], where the catalysts were used for C–O hydrogenolysis reactions. According to the data of temperature-programmed reduction, CO adsorption, XRD and XAFS, the catalyst with larger Mo amount contains Rh metal particles with size of ∼3 nm, and MoO_*x*_ species with average valence of around 4 are present on the surface of Rh metal particles under reductive conditions. It should be noted that we obtained essentially the same characterization results for different lots of Rh–MoO_*x*_/SiO_2_ (Mo/Rh = 1/8) catalysts [24, 30, 34], suggesting the good reproducibility in preparation of Rh–MoO_*x*_/SiO_2_ catalysts. Similar structures of unsupported Rh/Mo catalysts have been reported in the literature [13]: aggregates of Rh metal particles with the size of 2–4 nm and molybdenum oxide species whose valence is predominantly 4. The reaction mechanism over Rh–MoO_*x*_/SiO_2_ catalyst can be the same as that over unsupported Rh/Mo catalysts.

Several literature studies [6] proposed the reaction mechanism of hydrogenation of amides over bimetallic catalysts as follows: first, the carbonyl group of the amide is hydrogenated (equation (4)), and then dehydration occurs to form imine intermediate (equation (5)). Hydrogenation of imine gives amine product (equation (6)). The hydrogenation of deactivated carbonyl group (equation (4)) is the rate-determining step.4
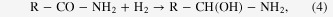

5
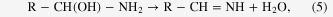

6
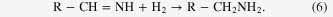



There is another reaction mechanism proposed in the literature: first amide is dehydrated to form nitrile (equation (7)), and then nitrile is hydrogenated to amine (equation (8)) [6, 14].7
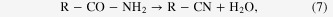

8
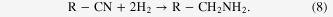



The present data agree with the former mechanism with imine intermediate (equations (4)–(6)). The positive reaction order with respect to hydrogen pressure (figure 5) indicates that the rate-determining step involves hydrogen species. For the mechanism with imine intermediate, the reaction order corresponded with that the step of equation (4) is rate determining. On the other hand, for the mechanism with nitrile intermediate (equations (7) and (8)), the reaction order means that the dehydration step (equation (7)) is fast. However, cyclohexaneacetonitrile, which is the dehydration product of cyclohexanecarboxamide, was not detected in the hydrogenation of cyclohexanecarboxamide. Although the concurrent participation of both mechanisms is not ruled out, the main reaction route should be the former mechanism.

Now we discuss the mechanism of addition effects of CeO_2_. As shown in section 3.2, two promoting effects were present: increase in the catalytic activity (substrate conversion) and increase in the selectivity to primary amine (target product). The former effect can be explained by the increase of the number of active site. According to the reported density functional calculation for Pt–ReO_*x*_/TiO_2_-catalyzed hydrogenation of amide [17], the amide substrate is first adsorbed on the Re^*n*+^ center with the carbonyl group, and then the carbonyl group is reduced. In contrast, as shown in our previous papers, the active sites of *M*^1^–*M*^2^O_*x*_/SiO_2_ catalysts (*M*^1^ = Rh, Ir; *M*^2^ = Mo, Re) for activation of alcohols in C–O hydrogenolysis are *M*^2^–OH sites [25, 26, 41–44], and the addition of solid acid to Ir–ReO_*x*_/SiO_2_ increases the number of Re–OH sites by protonation of Re–O^−^ [31]. The addition of solid base (CeO_2_) to Rh–MoO_*x*_/SiO_2_ may well decrease the number of acidic Mo–OH sites (equation (9)).9
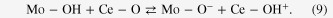



The Mo–OH site has Br⊘nsted acidity and thus the amide substrate can be adsorbed on the proton rather than the Mo^4+^ center which activates carbonyl group. Therefore, the addition of CeO_2_ to Rh–MoO_*x*_/SiO_2_ can increase the number of site for activation of carbonyl group of amide to increase the activity.

The latter effect (increase in the selectivity) is accompanied by the suppression of secondary amine formation. According to the literature, secondary amine is mainly produced by addition reaction of imine intermediate with primary amine (equation (10)) and addition of ammonia to the reaction media is effective to suppress secondary amine formation [13]. The reaction of equation (10) competes with the hydrogenation of imine (equation (6)). Suppression of the reaction of equation (10) and/or promotion of the reaction of equation (6) increases the selectivity to primary amine.10
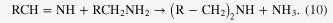



In the present system, ammonia was generated by alcohol (CyCH_2_OH) formation. One explanation of addition effect of CeO_2_ to increase selectivity is that basic CeO_2_ reduces the acidity of Rh–MoO_*x*_/SiO_2_ surface to increase the concentration of free ammonia in the reaction media (equation (11)).11
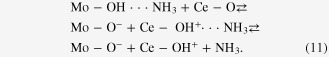



Another explanation for the increase in selectivity is that the step of imine hydrogenation (equation (6)) is accelerated by the CeO_2_ addition. From table 2, the systems that showed higher yield of reduction products (CyCH_2_NH_2_ + CyCH_2_OH + (CyCH_2_)_2_NH) tend to show higher selectivity ratio of CyCH_2_NH_2_/(CyCH_2_)_2_NH. Further investigation is necessary to clarify the mechanism of increasing selectivity to amine.

## 4. Conclusions

The addition of CeO_2_ to Rh–MoO_*x*_/SiO_2_ increases the catalytic activity in hydrogenation of cyclohexanecarboxamide to aminomethylcyclohexane. The selectivity to aminomethylcyclohexane is also increased by the addition of CeO_2_. The activity of this combined catalyst system is higher than that of unsupported Rh/Mo catalyst system, which has been reported in the literature, although the aminomethylcyclohexane yield is still lower. The crystallinity of CeO_2_ does not affect the addition effect, suggesting that only the weakly-basic nature of CeO_2_ surface induces the addition effect. The addition effect of CeO_2_ can be related to the ratio of Mo–O^−^ to Mo–OH sites on the surface of Rh–MoO_*x*_/SiO_2_.
